# Evaluation of surgical techniques in survival rate and complications of zygomatic implants for the rehabilitation of the atrophic edentulous maxilla: a systematic review

**DOI:** 10.1186/s40729-023-00478-y

**Published:** 2023-05-17

**Authors:** Peer W. Kämmerer, Shengchi Fan, Carlos Aparicio, Edmond Bedrossian, Rubén Davó, Dean Morton, Gerry M. Raghoebar, Sepehr Zarrine, Bilal Al-Nawas

**Affiliations:** 1 Department of Oral and Maxillofacial Surgery–Plastic Operations, University Medical Center Mainz, Augustusplatz 2, 55131 Mainz, Germany; 2 Shanghai Key Laboratory of Stomatology & Shanghai Research Institute of Stomatology, School of Medicine Second Dental Clinic, Ninth People’s Hospital, National Clinical Research Center for Oral Disease, College of Stomatology, Shanghai Jiao Tong University, 200011 Shanghai, China; 3 Indiana University School of Dentistry, Indianapolis, USA; 4 Zygomatic Unit at Hepler Bone Clinic, ZAGA Center Barcelona, Barcelona, Spain; 5 Department of Restorative Dentistry, School of Dentistry, University of Washington, Seattle, USA; 6 Department of Implantology and Maxillofacial Surgery, Vithas Davó Instituto Dental, Alicante, Spain; 7 Department of Prosthodontics, Indiana University School of Dentistry, Indianapolis, USA; 8 Department of Oral and Maxillofacial Surgery, University Medical Center Groningen and University of Groningen, Groningen, The Netherlands; 9 Private Practice, Saint Die, France

**Keywords:** Zygomatic implant, Maxillary atrophy, Maxillary defect, Technique, Survival, Complications

## Abstract

**Purpose:**

To assess the outcome [zygomatic implant (ZI) survival] and complications of the original surgical technique (OST) and an Anatomy-Guided approach (AGA) in the placement of ZI in patients with severely atrophic maxillae.

**Methods:**

Two independent reviewers conducted an electronic literature search from January 2000 to August 2022. The inclusion criteria were articles reporting at least five patients with severely atrophic edentulous maxilla undergoing placement OST and/or AGA, with a minimum of 6 months of follow-up. Number of patients, defect characteristics, number of ZI, implant details, surgical technique, survival rate, loading protocol, prosthetic rehabilitation, complications, and follow-up period were compared.

**Results:**

Twenty-four studies comprised 2194 ZI in 918 patients with 41 failures. The ZI survival rate was 90.3–100% in OST and 90.4–100% in AGA. Probability of complications with ZI with OST was as follows: sinusitis, 9.53%; soft tissue infection, 7.50%; paresthesia, 10.78%; oroantral fistulas, 4.58%; and direct surgical complication, 6.91%. With AGA, the presenting complications were as follows: sinusitis, 4.39%; soft tissue infection, 4.35%; paresthesia, 0.55%; oroantral fistulas, 1.71%; and direct surgical complication, 1.60%. The prevalence of immediate loading protocol was 22.3% in OST and 89.6% in the AGA. Due to the heterogeneity of studies, statistical comparison was only possible after the descriptive analysis.

**Conclusions:**

Based on the current systematic review, placing ZI in severely atrophic edentulous maxillae rehabilitation with the OST and AGA is associated with a high implant survival rate and surgical complications within a minimum of 6 months follow-up. Complications, including sinusitis and soft tissue infection around the implant, are the most common. The utilization of immediate loading protocol is more observed in AGA than in OST.

**Graphical Abstract:**

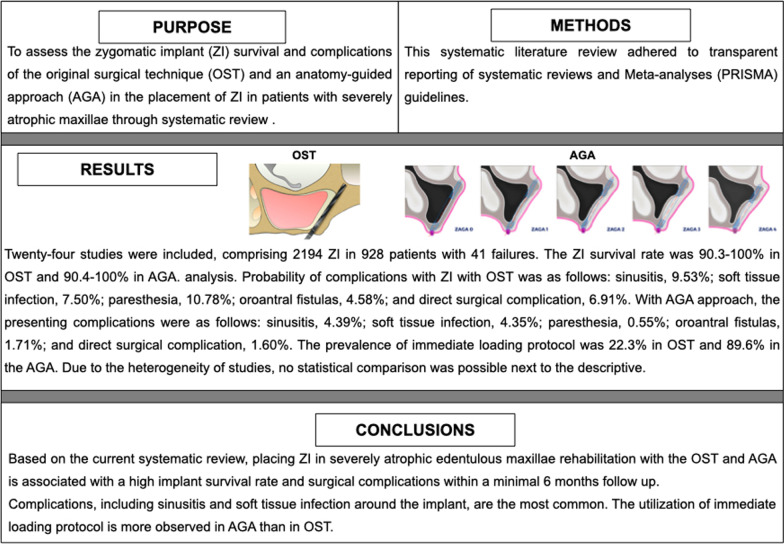

## Background

Zygomatic implant (ZI) was introduced systematically in 1988 to rehabilitate patients who had undergone tumor and maxillectomy by Brånemark. Later, a new concept was proposed of utilizing long implants to an anchorage in zygomatic bone for the edentulous maxilla [1]. The result of a total of 52 ZIs with a 96% success rate with over 5 years of follow-up was reported and considered an alternative technique to avoid massive bone grafting before implant placement. The classic Brånemark approach was a two-stage procedure with 2 ZIs placed in premolar/molar and combined with 2 to 4 regular implants (RIs) placed in the anterior area for delayed restoration [2]. Since 2006, Bedrossian and Chow et al. have proven the reliability of immediate loading and function of ZIs, the protocol of immediacy of ZI has been widely investigated and brought great benefit to the patient compared with traditional grafting procedures [3, 4]. Later, the classic approach was further modified to the so-called ‘quad approach’, which indicated the severely atrophic maxilla with neither sufficient bone in the anterior and posterior zone for placement of conventional dental implants and for placing 2 ZI on each side of the zygoma instead [5].

A sizeable lateral osteotomy to the sinus is prepared in the original surgical technique (OST) from Brånemark. From a prosthetic point of view, the optimal entrance was as far posterior and close to the crestal midline as possible. These combined considerations usually meant that the fixture originated from the second premolar region [2]. This often led to the implant’s platform emerging palatal to the crestal ridge following the zygomatic alveolar crest into the sinus and engaged in the zygoma [6].

In the following years, the original technique has been further elaborated by many clinicians regarding the sinus position and the crestal emergence to allow for better individual anatomical and prosthetic adaptation [3, 7–10]. This has led to various modifications and definitions of OST, in which one major part is related to the sinus anatomy. Stella and Warner modified it to a sinus-slot technique which avoids sinus window formation and lifts the sinus membrane for placement of zygomatic implants in patients with extreme buccal concavities in the maxillary sinus area [11]. This slot results in a smaller antrostomy that will orient the twist drills for implant placement. In 2003, Boyes-Varley and colleagues altered the OST to establish improved surgical site access and reduce postoperative morbidity. They also changed implant head angulation into a 55° correction [12] or described ZI as a rescue implant in failed anterior or posterior tilted implants [13]. In 2008, Malo and his team proposed an extra-maxillary approach by preparing ZI trajectory exclusively in the zygomatic bone and allocated it in the groove of the lateral wall of the maxilla to avoid sinusitis [9]. Another aspect, which is not always reported clearly in clinical publications, is related to crestal reduction as performed by some clinicians [14].

In 2010, the classification of the zygomatic anatomy-guided approach (ZAGA) was described by Aparicio based on a cross-sectional study of 200 human radiographic sites [15]. This approach was organically introduced to refine “Anatomy-Guided” techniques for different anatomical solutions with the flat maxillary wall to the concave or atrophied maxillae. By following specific prosthetic, biomechanical, and anatomical factors, establishing the entrance point depends on the vertical and horizontal resorption of the alveolar/basal process and the anterior maxillary wall curvature. After years, this classification has been broadly used in teaching and clinical decision-making [16]. The authors know that no precise discriminative definition of the OST and the “Anatomy-Guided” procedure exists. Although the OST was a rather generic description of zygomatic implant position, in recent publications defined zygomatic implant positions for different anatomical situations are suggested (“Anatomy-Guided” techniques. Current systematic reviews were primarily aimed at comparing the survival rates with ZI treatment in different levels of atrophy maxillae, such as the classic approach versus the quad approach or ZI rehabilitation versus regular implant restoration [17, 18].

On the other hand, these descriptions are broadly used, and it is time to understand if these approaches have different tangible outcomes. However, various techniques have reported complications, such as sinus infections, intra-oral soft tissue infections, nerve disturbances, oroantral fistula, extra-oral hematoma, and prosthetic complications [19, 20]. As there is no systematic literature review comparing the OST and Anatomy-Guided approaches, the purpose of the present investigation was to compare both surgical techniques regarding ZI survival and complication rate through a systematic review.

## Methods

This systematic literature review adhered to Transparent Reporting of Systematic Reviews and Meta-Analyses (PRISMA) guidelines [21].

### PICO question

The focused PICO (Population, Intervention, Comparison, Outcome) question was:

“In patients with acquired or congenital disabilities or atrophy of the maxilla (P) insertion of zygomatic implants (I) of which of the two surgical techniques (OST and Anatomy-Guided; C) is more predictable in implants survival (O)?

For the secondary research question, complication rates and implant-related quality of life were compared between the two techniques.

### Search strategy

The systematic search was conducted on PubMed MEDLINE, SCOPUS, and Web Of Science databases using relevant terms for the focused question. The used search terms were as follows: (“zygomatic” OR “zygoma” OR “zygomaticus”) AND (“dental implant” OR “dental implants”) NOT “animal” NOT “cadaver”.

The search period was from January 2000 to August 2022. The target was human studies published in English or German language. The searched database modified the search strategy and terms.

### Inclusion criteria

**Fig. 1:**
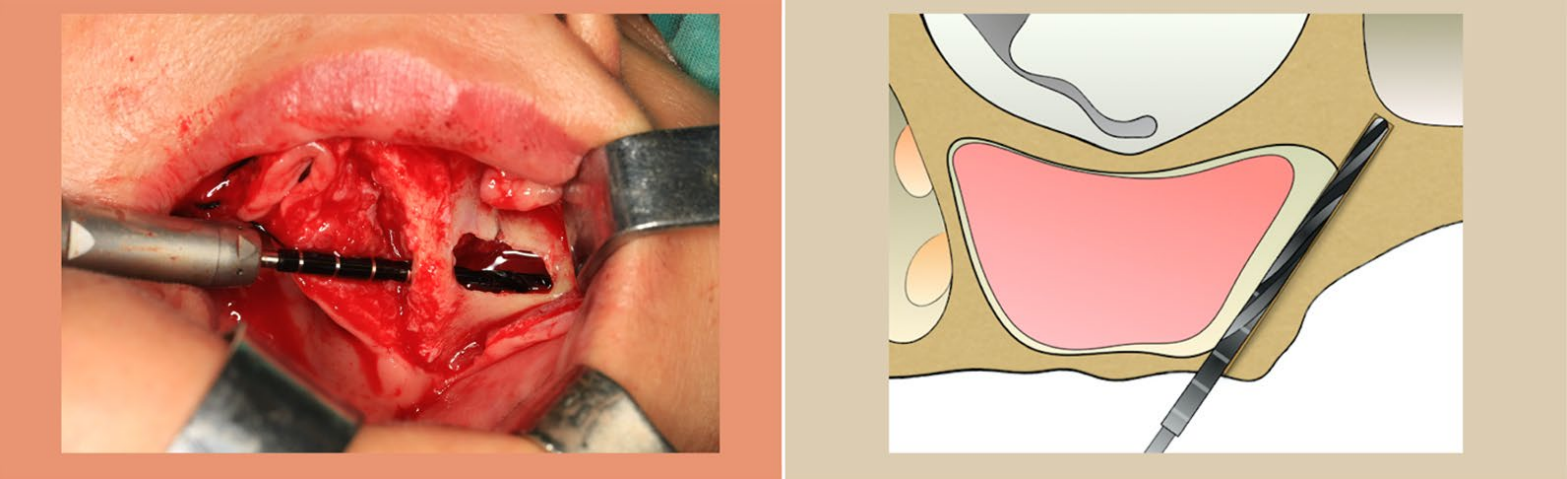
The original surgical technique described by Branemark (OST) begins with a Le Fort I-type incision. A full-thickness mucoperiosteal flap is elevated to provide direct visualization of the trajectory of the implant from the premolar/molar region of the alveolar bone to the zygoma body. The dissection is continued from the lateral wall of the maxilla towards the zygomatic bone to allow for increased visibility of the zygomatic region and the infraorbital nerve. A lateral window of approximately 10 * 5 mm is then made into the lateral aspect of the maxillary sinus using a round bur (**A**). Once the membrane has been exposed, it should be carefully elevated medially and superiorly. The entrance of the ZI is marked with a round bur on the palatal side of the crest. The drilling sequence starts at the alveolar ridge, passing through the maxillary sinus, and the drill is advanced to reach the body of the zygoma to the desired emergence level (**B**) [2] (Figure provided by Yiqun Wu)

**Fig. 2:**
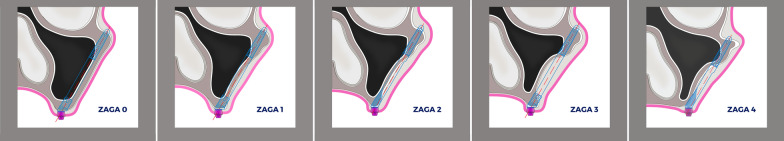
For Anatomy-Guided as an evolution of the extra-sinus approach, the relationship of the zygomatic buttress–alveolar crest area is classified into five different types. In this technique, the path of the ZI body can range from total intra-sinus (ZAGA 0) to the wall of the maxilla (ZAGA 1 & 2) to total extra-maxillary sinus (ZAGA 3 & 4). The curvature of the external wall of the maxillary buttress determines the final relationship between the implant and the anterior maxillary wall. For surgical access, a slightly beveled palatal incision starts from the posterior buccal aspect of the maxillary tuberosity to the midline. According to the prosthodontics aspect, the starting point (implant head emergence) should be at or close to the top of the alveolar ridge crest. When the residual bone at the sinus floor level has adequate thickness and width (minimum: 4 mm height, 6 mm width) in a patient without a history of periodontitis, the position of the entry point should be close to the middle portion of the crest with an intra-sinus starting path of the implant if the maxillary wall is flat or convex. When the crestal bone height or thickness is inadequate, the alveolar entrance point should be shifted to the buccal, regardless of the maxillary wall curvature. Based on the maxillary wall concavity and the height of the new bone, the osteotomy is shaped like a tunnel or canal [16, 22]

- studies aimed at investigating patients with atrophic upper jaws rehabilitated with ZIs;
- studies used and demonstrated the OST technique (Fig. 1) and/or Anatomy-Guided or so-called “ZAGA” technique (Fig. 2) in method and/or results with discretions and/or citations and/or tables;
- clinical studies in humans, including RCT, prospective, retrospective, and case series studies;
- a minimum of 5 patients followed for at least 6 months;
- must specify the number of participants, implants, follow-up duration, failures, survival rate, and complications.

### Exclusion criteria

- articles published in another language other than English or German;
- topic not relevant to the focus questions;
- reviews, systematic reviews;
- case reports with less than five patients and or follow-up of fewer than 6 months, technical notes;
- animal studies, in vitro studies;
- insufficient participant information and no response from investigators when seeking clarification;
- previous investigations reporting on the same patient population (excluded but retained for reference).

### Study selection and quality assessment

Quality assessment, according to PRISMA, was aimed for.

### Data extraction

Two reviewers (PWK, SCF) independently screened titles and abstracts of all studies retrieved from the search mentioned above strategy and voted for inclusion or exclusion, respectively. Conflicts were resolved in discussion with a third reviewer (BA). Subsequently, full-text screening was performed, and studies were excluded when they failed to meet the inclusion criteria or fell into the category of exclusion criteria.

The following data were extracted from each study:

- study designs: randomized/nonrandomized controlled trial, prospective study, retrospective study, case series report;
- the characteristic of patients, follow-up period;
- number of ZI, length of ZI, number of RI, successful rate, complication, survival rate, approaches, ZI’s brand;
- any ZI-related complications.

## Results

### Paper selection process

**Fig. 3:**
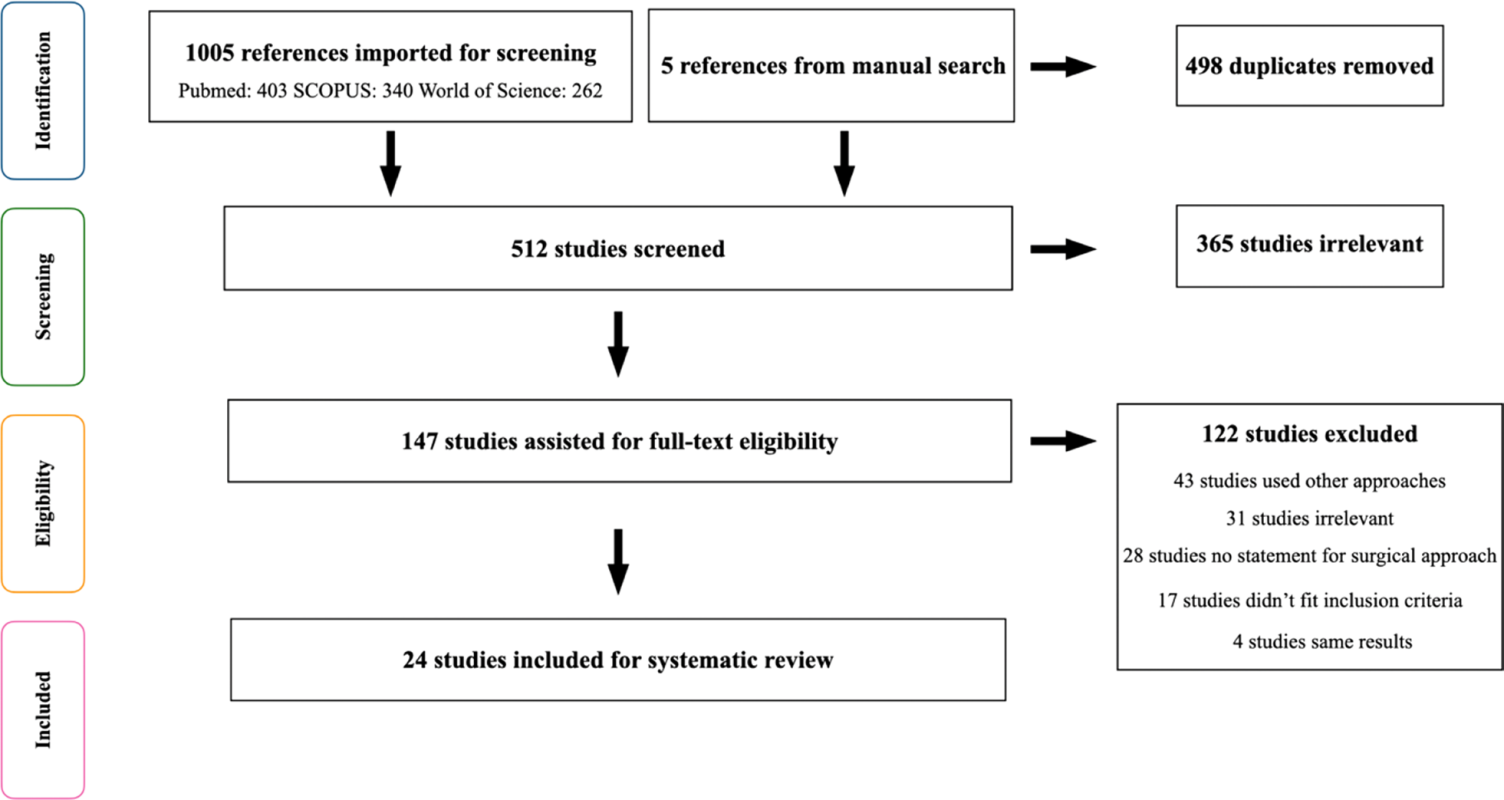
PRISMA flow diagram

One thousand and five articles were identified through Med MEDLINE, SCOPUS, and Web of Science databases. After analyzing the titles and abstracts and identifying duplicate publications, 863 articles were excluded, leaving 147 for further review. In 2010, 2013, and 2015, Davó et al. reported 3 results at different follow-up times of the same study population [23–25]. In 2014, Aparicio et al. reported the results of OST and Anatomy-Guided techniques, of which the OST technique was already included in a previous study [26]. In 2004, Hirsch et al. [27] reported the results of a 1-year follow-up of the same population as Kahnberg’s study [28]. Five studies, along with a manual search, were also included. The inclusion and exclusion criteria were applied, and 24 articles were considered acceptable for full-text analysis [2, 4, 7, 14, 19, 25, 28–45] (Fig. 3).

Descriptive data of the 24 studies included in the systematic review are shown in Table 1 in 2 subgroups (OST and Anatomy-Guided).

**Table 1:** Descriptive analysis of the included articles (type of ZI insertion, authors, date of publication, type of study (P = prospective, R = retrospective RCT = randomized clinical trial), number of patients, age and gender of patients, follow-up period, degree of maxillary atrophy, partially/totally edentulous maxilla, number, length, brand, and survival of ZI, approach and planning software)

Original surgical technique (OST)														
Authors	Published	Type of study	Patients [*n*]	Patients’ average age (range) [years]	Gender	Mean follow-up period (range) [months]	Degree of maxillary atrophy	Partially/totally edentulous maxilla	Zygomatic implant (ZI)				Approach (patient [*n*])	Planning software
									No. of ZI [*n*]	ZI average length (range) (mm)	ZI’s brand	ZI’s Survival rate (failed implant)		
Aleksandrowicz [29]	2019	R	22	40.40 (36–69)	M11 F11	NM (50–152)	NM	Partially/totally	24	NM (30–45)	Nobel Biocare	95.8% (1)	1 ZI (12) 2 ZIs (8) 3 ZIs (1) Quad (1)	NM
Aparicio [19]	2014	P	22	53.81 (NM)	M8 F14	135.2 (NM)	Residual bone height and width less than 4 mm	Partially/totally	41	NM (30–50)	Nobel Biocare	95.12% (2)	Unilateral (NM) Classic (NM)	NM
Becktor [30]	2005	R	16	61.1 (29–77)	M6 F10	46.4 (9–69)	C&H	Totally	31	42.25 (30–50)	Nobel Biocare	90.3% (3)	Classic (27) Quad (4)	Nobel Clinician
Branemark [2]	2004	R	28	58.3 (39–79)	M12 F16	NM (60–120)	L&Z D, E	Partially/totally	52	NM	BOC & Exopro	94% (3)	Unilateral (4) Classic (24)	NM
Chow [4]	2006	P	5	49.8 (43–60)	M4 F1	10 (NM)	NM	Totally	10	49.5 (45–52.5)	Nobel Biocare	100% (0)	Classic (5)	SimPlant
Davo [7]	2007	R	18	58 (44–74)	M6 F12	14 (6–29)	NM	Totally	36	46.59 (40–52.5)	Nobel Biocare	100% (0)	Classic (18)	NM
Davo [31]^a^	2008	R	26	NM	NM	21.9 (13–42)	L&Z B, C	Partially/totally	61	NM	Nobel Biocare	100% (0)	1 ZI (4) Classic (30) Quad (2)	NM
Davo [32]	2009	R	24	51.4 (36–72)	M8 F16	60 (NM)	NM	Partially/totally	45	45.38 (40–50)	Nobel Biocare	97.4% (1)	1 ZI (1) Classic (23)	NM
Duarte [33]	2007	P	12	NM	NM	30 (NM)	Residual bone height and width less than 2 mm	Totally	48	NM	Nobel Biocare	95.8% (2)	Quad (12)	NM
Fernandez [34]	2014	R	80	55.5 (25–75)	M40 F40	27 (6–48)	NM	Partially/totally	244	NM	NM	99.6% (1)	Classic (79) Quad (1)	NM
Kahnberg [28]	2007	P	76	58 (35–77)	M19 F57	36 (NM)	NM	NM	145	NM	NM	96.3% (5)	Classic (76)	NM
Malevez [35]	2004	R	55	M 62 (40–76) F57 (22–79)	M14 F41	NM (6–48)	Residual bone height less than 5 mm	Totally	103	42.69 (35–50)	Nobel Biocare	100% (0)	1 ZI (6) 2 ZIs (49)	NM
Stiévenart [36]	2010	R	20	56 (35–75)	M1 F19	NM (6–40)	L&Z D, E	Totally	80	NM (30–52.5)	Nobel Biocare	96% (3)	Quad (20)	Procera software

Anatomy-guided (AGA)														
Authors	Published	Type of study	Patients [*n*]	Patients’ age range (average) (years)	Gender	Follow-up period range (average) (months)	Degree of maxillary atrophy	Partially/totally edentulous maxilla	Zygomatic implant (ZI)					
									No. of ZI [*n*]	ZI average length (range) (mm)	ZI’s brand	ZI’s survival rate (failed implant)	Approach (patient [*n*])	Planning software
Aparicio [19]	2014	P	80	63.1 (NM)	M25 F55	55.4 (NM)	Residual bone height and width less than 4 mm	NM	156	NM (30–50)	Nobel Biocare	96.79% (5)	NM	NM
Aparicio [37]	2022	R	20	59.2 (NM)	M11 F9	18.8 (12–28)	Bedrossian classification	NM	59	NM	Straumann		Classic (10) 3 ZIs (1) Quad (9)	DTX Studio Implant software
Atalay [38]^a^	2017	R	10	55.2 (23–68)	M5 F5	28 (6–96)	C&H IV, V, VI	Totally	21	NM (30–47.5)	Nobel Biocare	90.4% (2)	Classic (9) 3 ZIs (1)	NM
Chana [39]	2019	R	43	NM (42–88)	M22 F23	90 (NM-216)	NM	NM	80	NM (20–52.5)	Nobel Biocare	94.32% (5)	NM	Nobel Clinician
Davo [25]	2015	P	17	57.7 (41–78)	M7 F10	NM (36–60)	C&H IV, V, VI	Totally	68	45.36 (35–52.5)	Nobel Biocare	100% (0)	Quad (17)	Procera Software
Davó [40]	2020	R	82	57 (33–78)	M29 F53	10.5 (0–29)	L&Z	Partially/totally	182	41.85 (30–52.5)	Nobel Biocare	100% (0)	3 ZIs (3) Classic (62) Quad (16)	NM
Fernández-Ruiz [41]	2021	RCT	40	60.18 (NM)	M17 F23	19.40 (12.00–22.00)	NM	Totally	139	40.03 (30–60)	Sarria	100% 0)	Classic (NM) Quad (NM)	NM
Nave [42]	2020	R	102	NM	NM	38 (12–144)	NM	Partially/totally	206	NM	Nobel Biocare Neodent Instradent	97.57% (5)	1 ZI (27P) 2 ZIs (57P) 3 ZIs (3P) NM (1P)	NM
Penarrocha-Diago [43]	2021	R	19	61.7 (54–73)	M3 F16	20.1 (12–41)	C&H V, VI	NM	31	NM	Nobel Biocare Sarria	100% (0)	NM	/
Wang [14]	2021	P	15	47.2 (19–71)	M3 F12	17.2 (12–36)	C&H IV	Totally	60	48.68 (35–52.5)	NM	100% (0)	Quad (15)	Nobel Clinician
Wu [44]^a^	2022	R	61	46.8 (18–79)	M33 F28	24.11 (NM)	Residual bone height less than 3 mm	Totally	188	NM	Nobel Biocare	98.4% (2)	Classic (26) Quad (35)	Nobel Clinician VectorVision2
Zhao [45]	2018	R	25	47.1 (19–69)	M12 F13	23.00 (NM)	Residual bone height less than 3 mm	Totally	84	48.75 (40–52.5)	Nobel Biocare	98.0% (1)	Classic (NM) Quad (NM)	Nobel Clinician

*NM* not mentioned, *C&H* Cawood–Howell classification, *L&Z* Lekholm and Zarb classification, *R* retrospective study, *P* prospective study, *RCT* randomized controlled trials

^a^Data were partially excluded due to use other techniques but OST or ZAGA

### General property of the studies included

The articles were described according to the surgical technique of zygomatic implant placement, follow-up time, implant survival rate, complications, type of prosthesis, and loading protocol. The main characteristics of the studies included are described in Tables 1 and 2 according to the study model, patients, number of regular and zygomatic implants, loading, prosthetic rehabilitation, complication, and follow-up time. A total of 17 retrospective studies, 6 prospective studies, and 1 RCT were retrieved from the search.

**Table 2:** Descriptive analysis of the included articles (type of ZI insertion, prosthesis type, loading protocol with number of patients, surgical complications, sinusitis, soft tissue infection, paresthesia/pain, oroantral fistula, prosthesis complication)

Original surgical technique									
Authors	Prothesis type	Loading protocol (immediately/delayed)	Patients (*n*)	Complication					
				Surgical complication [p]	Sinusitis [P]	Soft tissue infection	Paresthesia/pain (temporally/present) [P]	Oroantral fistula formation [P]	Prosthesis complication [cases]
Aleksandrowicz [29]	Fixed	Delayed	22	NM	4	3ZIs^b^	NM	NM	NM
Aparicio [19]	Fixed	Delayed	22	6 facial hematoma 5 lip laceration	6	1P	6 (temporally)	3	4 acrylic fractured 25 porcelain fractured 2 framework fractured 6 screws fractured 9 screws/abutment loosening
Becktor [30]	Fixed	Delayed	16	0	6	9P	NM	5	0
Branemark [2]	Fixed	Delayed	28	0	4	2P	NM	NM	NM
Chow [4]	NM	Immediately	5	NM	NM	NM	NM	NM	0
Davo [7]	Fixed	Immediately	18	0	1	NM	NM	NM	NM
Davo [31]^b^	Fixed	Immediately	26	NM	0	NM	NM	0	NM
Davo [32]	Overdenture fixed	NM	24	0	5	NM	NM	0	NM
Duarte [33]	Fixed	Immediately	12	NM	0	NM	NM	0	NM
Fernandez [34]	Fixed	Delayed	80	2 subcutaneous malar emphysema	6	NM	1 (NM)	2	NN
Kahnberg [28]	Fixed	Delayed	76	NM	1	3P	2 (temporally)^a^ 1 (present)^a^	5^a^	9
Malevez [35]	Fixed	Delayed	55	NM	5	0	NM	0	1 esthetic problems
Stiévenart [36]	Fixed overdenture	Delayed immediately	20	NM	1	3P	1 (NM)	NM	0
Total									56
No. of patients reported (reported cases)	414			188 (13)	409 (39)	240 (18)	102 (11)	327 (15)	
Total incidence rate^b^				6.91%	9.53%	7.50%	10.78%	4.58%	

Anatomy-guided technique									
Authors	Prothesis type	Loading protocol (immediately/delayed)	Patients (*n*)	Complication					
				Surgical complication	Sinusitis	Soft tissue infection	Paresthesia	Oroantral fistula formation	Prosthesis complication
Aparicio [19]	Fixed	Immediately	80	1 facial hematoma	3	0	0	2	65 acrylic fractured 2 porcelain fractured 7 screws fractured 16 screws/abutment loosening
Aparicio [37]	Fixed	Immediately	20	0	1	2P	NM	NM	NM
Atalay [38]^b^	Fixed	Delayed	10	0	0	1P	0	0	0
Chana [39]	Removable fixed	Delayed immediately	45	0	3	4P	NM	NM	8 abutments loosening
Davo [25]	NM	Immediately	17	1 orbital cavity penetrated	2	4P	NM	1	1 abutment screw fractured 2 prostheses fractured
Davó [40]	Fixed	Delayed immediately	82	NM	5	1P	0	0	NM
Fernández-Ruiz [41]	Fixed	Immediately	40	1 orbital cellulitis	1	21ZIs^b^	0	0	0
Nave [42]	Fixed	Immediately	102	NM	5	2P	NM	2	NM
Penarrocha-Diago [43]	NM	Delayed immediately	19	0	0	0	0	1	NM
Wang [14]	Fixed	Delayed immediately	15	2 facial hematoma with lip laceration	0	3P	1 (temporally)	NM	3 screw loosening and temporary prothesis fractured
Wu [44]^b^	Fixed	Immediately	61	Navigation system-related complications	NM	NM	NM	NM	NM
Zhao [45]	Fixed	Immediately	25	NM	0	NM	NM	NM	NM
Total reported cases				5	20	17	1	6	104
No. of patients reported (reported cases)	516			311 (5)	455 (20)	390 (17)	181 (1)	350 (6)	
Total incidence rate^b^				1.60%	4.39%	4.35%	0.55%	1.71%	

*NM* not mentioned, *P* patient

^a^Data extracted from results of 1 year followed up in 2004 of the same study population in 2007

^*^Data were excluded from results because not reported the exact patient number

### Study characteristics and risk of bias assessment

Agreement between the two reviewers was determined for the inclusion or exclusion of reports as only one comparative study focused on OST and Anatomy-Guided; a meta-analysis was not performed. Descriptive statistics were pooled to report and compare the data. Statistical heterogeneity between all the studies included in this systematic review was not assessed because all the studies had a different number of patients, observational periods, and descriptive methods, making a statistical comparison impossible.

### ZI survival rate

The final selection included 24 studies reporting on using OST and/or Anatomy-Guided technique while treating 918 atrophic resorption maxillae patients via a total of 2194 implants [2, 4, 7, 14, 19, 25, 28–45]. The survival rate of ZI was defined as the implant remind in the zygoma and alveolar, which was functional.

For the OST technique, 13 studies (9 retrospective and 4 prospective) were included with 404 patients and 920 Zis (Tables 1 and 2). This technique's survival rates of ZI ranged between 90.3 and 100%. In 404 patients, 206 patients received 2 ZIs with RIs as classic approach, 40 patients received 4 ZIs as quad approach and 85 patients received 1 to 3 ZI in unilateral or bilateral zygoma, others were not fully reported or not mentioned. The ZI failed due to rotational mobility, sinusitis, infection, or implant malposition was recorded in 21 cases.

For the Anatomy-Guided technique, 12 studies (8 retrospective, 3 prospective, and 1 RCT) were included with 514 patients and 1274 Zis (Tables 1 and 2). The survival rates of ZI from Anatomy-Guided ranged between 90.4 and 100%. Of 514 patients, 107 patients received 2 ZIs with RIs as classic approach, 92 patients received 4 ZIs as quad approach and 203 patients received 1 to 3 ZI in unilateral or bilateral zygoma, others were not fully reported. The ZI failed due to rotational mobility, fracture, or infection was recorded in 20 cases.

### Loading protocol (immediate/delayed)

The two loading protocol information are extracted in Table 2, and all studies reported the type of loading protocol except one from Davo [32]. Among these 23 studies, 16 studies (5 from OST and 11 from the Anatomy-Guided technique) evaluated the use of ZI with immediate function protocols, and the high survival of ZIs was reported.

In the OST group, seven studies used a delayed protocol, 4 used an immediate loading protocol, and 1 used both protocols for loading. The prevalence of loading protocols of OST was 77.7% (680/875) for delayed loading and 22.3% (195/875) for immediate loading. For the Anatomy-Guided technique, 1 study used a delayed protocol, 7 used immediate loading protocols, and four used both protocols. Accordingly, the prevalence of loading protocols of Anatomy-Guided was 10.4% (132/1274) for delayed loading and 89.6% (1142/1274) for immediate loading.

Concerning the impact of loading protocols on ZI failure, the failure rate of OST was 2.2% (15/680) in the delayed loading group and 2.56% (5/195) in the immediate loading group. The failure rate of Anatomy-Guided was 1.51% (2/132) in the delayed loading group and 1.75% (20/1142) in the immediate loading group (Table 2).

### Complications

Details of complications are described in Table 2. Pooled incidence rates from the OST technique were 9.53% for sinusitis, 7.5% for soft tissue infection, 10.78% for paresthesia, 4.58% for oroantral fistula formation, 6.91% for surgical-related complications, and 56 incidents for prosthesis-related problems. Pooled incidence rates from the anatomy-guided technique were 4.39% for sinusitis, 4.35% for soft tissue infection, 0.55% for paresthesia, 1.71% for oroantral fistula formation, 1.6% for surgical-related complications, and 104 incidents for prosthesis-related complications. However, numbers may be underestimated in both techniques since most clinical studies have yet to report the presence or absence of complications (Table 2).

### ZAGA classification distribution

**Fig. 4:**
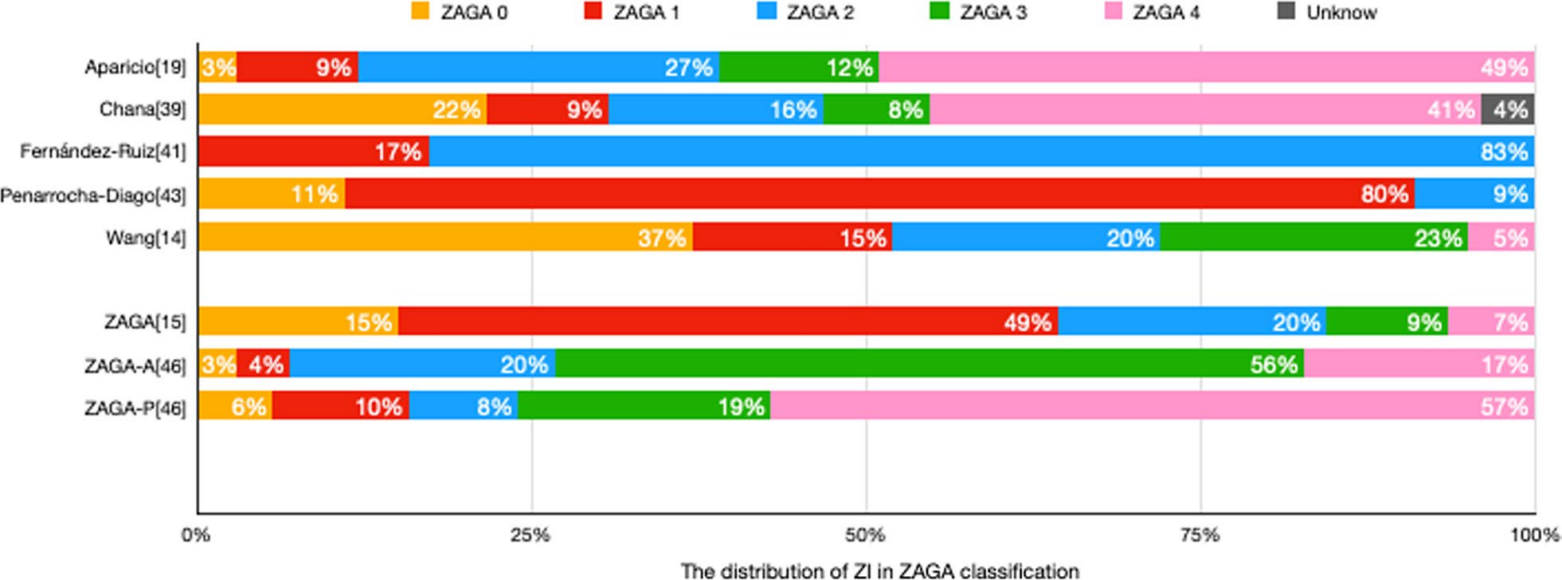
Distribution of the type of ZAGA classification between eligible studies [14, 37, 39, 41, 43] and ZAGA studies [15, 46]. ZAGA-A evaluated the anterior ZI’s distribution in the ZAGA classification, and ZAGA-P evaluated the posterior ZI’s distribution [46]

Five studies demonstrated the classification type of ZI position according to ZAGA (Fig. 1) [14, 37, 39, 41, 43]. In Aparicio’s original study, the 200 implants in five groups were classified from ZAGA 0 to 4, representing 15%, 49%, 20.5%, 9%, and 6.5%, respectively [15]. Moreover, his recent study modified the classification to evaluate the "Quad approach" with 488 ZI in anterior and posterior ZI positions [46]. The implants placed in the anterior maxilla (ZAGA-A) corresponding to each of the five osteotomy paths were 2.9% for type 0, 4.5% for type 1, 19.7% for type 2, 55.7% for type 3, 17.2% for type 4. Furthermore, an implant placed posteriorly was named from ZAGA type P-0 to P-4. The percentages for each class were as follows: 5.7% for type 0, 10.2% for type 1, 8.2% for type 2, 18.4% for type 3, and 57.4% for type 4. Two studies described the implant position at intra-sinus (type 0), wall of the sinus (type 1 & 2), and extra-sinus (type 3 & 4): Atalay et al. reported 95% intra-sinus and 5% extra-sinus of a total 21 ZIs placement [38], and Davo et al. described 5% of intra-sinus, 52% of the wall of the sinus and 42% of an extra-sinus pathway of 182 ZIs [40] (Fig. 4).

## Discussion

In this systematic review, the survival and complication rates of ZI were compared via original surgical technique (OST) and Anatomy-Guided approach (AGA) in patients with severely atrophic maxillae. Both techniques detected a high implant survival rate without related complications.

In 2004, Brånemark et al. published the first long-term follow-up study of ZI placement in the edentulous maxilla [2]. Two crucial pieces of information have been demonstrated in his report: first, to describe the OST for ZI insertion with a two-stage procedure through the maxillary sinus to an anchorage in zygoma bone; second, to evaluate the biomechanical model of the classic approach to support fixed restoration with ZI and regular implants. In 2014, Aparicio et al. compared the OST with ZAGA (Anatomy-Guided) to evaluate the long-term outcome of two surgical techniques and the incidence of complications [19].

The results of present review show that both procedures have similar clinical outcomes concerning implant survival. In brief, OST with 923 ZI had a survival of 90.3–100%, and 1302 ZI placed via Anatomy-Guided approach showed a survival of 90.4–100%. Compared to Chrcanovic’s systematic review, the 12-year cumulative survival rate was 95.21% with 4556 ZIs, and most failures were found at the early postoperative stages [47]. Compared to traditional implant treatment, the most remarkable advantage of using this graftless treatment is immediate loading to restore the patient’s oral function and esthetics after surgery. Different prevalence between delayed and immediacy protocols was found in OST (77.7% vs. 22.3%) and Anatomy-Guided technique (10.4% vs. 89.6%), respectively. Though this may be the result of more recent studies in cases of Anatomy-Guided techniques and advances in materials. The failure rate of immediate loading protocol in OST and Anatomy-Guided was 2.56% and 1.75%. Results were reported in an analysis of 103 failures collected from a review of 4566 ZIs, in which the failure rate of the immediate loading protocol was 1.7% [47]. However, even with the high survival rate evaluated, the surgical, biomedical, and prosthodontic complications should be well discussed.

### Sinusitis

ZI-associated sinusitis was the most frequent complication after ZI placement [47–51], even if a definition of diagnostic criteria or clinical implications is rarely reported. The ORIS criteria have been proposed by Aparicio et al., which evaluate the rhino-sinus status by comparison of pre-surgical and post-surgical CBCT and a clinical questionnaire [52]. The evidence that ZI placement may result in a foreign body reaction to the sinus membrane still needs to be discovered. Sinusitis could result from perforation of the Schneiderian membrane during the operation, the mobility from the ZI, the response of the operated sinus with whole blood, or the lack of osseointegration of the coronal part of ZI. In a comparative study, 27.2% and 3.7% of cases had reported sinusitis in OST and Anatomy-Guided groups, respectively.

Aparicio et al.’s comparative study showed significant differences between the two surgical approaches [19]. The Anatomy-Guided minimized the risk of pathology associated with the maxillary sinuses compared to the OST (76% vs 55% of patients with negative Lund Mackay and Lanza Kennedy tests). The present review (with incidence rates of 9.53% in OST vs. 4.39% in Anatomy-Guided) gave evidence for slightly different results. Therefore, it seems that the pathway of ZI, as well as the site of antrostomy might be a factor irritating the mucosa and/or obstructing the nasal complex with consequent sinusitis or influencing the preservation of the osseous/mucosal seal around the implant by preventing or favoring bacterial passage. However, 6 ZI had been reported to cause sinusitis in ZAGA 0–3 classification and 9 ZIs with sinusitis in ZAGA 4 & 5 [39, 40]; in the other 5 cases, the characteristics of implant position were not reported. A systematic review comparing the cumulative incidence of sinusitis in patients with ZI placed with an intra-sinusal pathway and extra-sinusal pathway also showed significant differences (7.2% vs. 1.8%) [18].

Accordingly, the preoperative evaluation of ZI treatment should include a clinical and radiology examination of the maxillary and other paranasal sinuses, especially for patients with a history of maxillary sinusitis. Although patients with a history of sinus clearance disturbing factors show no signs of sinus pathology at the time of surgery, they run a much higher risk of developing sinus pathology post-surgery. Pre-operative screening can be worthwhile. If such subject screening shows structural clearance disturbances, surgical intervention might be needed before placement of ZI is performed, even if there are no actual signs of sinus pathology. Patients with heavy smoking or untreated sinusitis should instead not undergo ZI treatment [53]. Few studies also evaluated the change of thickness of the Schneiderian membrane in CBCT. In one study, 12.2% of sinuses showed an ostium obstruction without clinical symptoms [45]. In another, 14 of 20 sinuses with diffuse membrane thickening had already presented the thickening in the preoperative scan [7].

### Local soft tissue infections

The infections of peri-implant soft tissue at the coronal part of the ZI could show as peri-implant mucosal hyperplasia and peri-implant mucosal recession with exposure to the implant surface or abutment [6]. So far, there is no consensus definition for peri-implantitis of ZI because the major anchorage part of the implant lies within the zygomatic bone. Peri-implant mucosal hyperplasia is mainly caused by improper oral hygiene maintenance around the abutment site. Therefore, pontic contours in fixed prostheses between the prosthesis' base to the ridge's crest are crucial [54]. A “channel gap” at the transition zone might be created in the prosthesis to permit floss threading for daily oral hygiene. 16 of 18 patients (88.8%) from eligible studies had reported mucosal hyperplasia around abutments from 5 studies in OST with the symptom of redness, swelling, suppuration, and oral hygiene problems [19, 28–30, 36]. The ORIS criteria evaluation explained that the palatal emergence of ZI will lead to the construction of bulky prostheses with an intra-sinus approach. If the distance between the offset of the abutment to the ridge is more than 15 mm, daily hygiene might be significantly more challenging to be performed [52]. On the other hand, 9 of 17 patients (52.9%) from eligible studies showed recessions at an implant-abutment level in the ZGAG technique [14, 32, 39, 42]. In cases of a severely atrophic maxilla (Cawood–Howell V & VI) [55] or an extra-sinus pathway (ZAGA 4 & 5) for placement ZI, this might lead to exposure of the buccal side of a rough surface neck without sufficient bone around.

For this reason, soft tissue usually shows insufficient or lack of keratinized tissue which tends to cause mild tissue recession. Chana et al. used xenografts and autogenous bone to cover exposed threads and performed additional grafting around osteotomy sites; here, a recession was noted in 4 of the 12 cases where grafting was performed and 11 of 73 without grafting [39]. In another, two groups were used for soft tissue grafting. The authors used guided bone regeneration (GRR) individually depending on the emergence of the implant (rough/smooth) and the state of the alveolar process [43]. Results showed no biological complications. Aparicio et al. used a new design of flat ZI with a machined surface after channel osteotomy, and the respective study reported 2 ZI with recessions in 1 patient [37]. However, the efficiency of tissue regeneration around the ZI neck needs more scientific evidence for long-term observation and evaluation.

### Fistula

The problem of the oroantral fistula is believed to be caused by the lack or lose of osseointegration between the severe atrophic alveolar bone and the marginal area around palatal placed ZI, which can result in a communication between the maxillary sinus and the oral cavity and might result in sinusitis. Thirteen cases and six were reported in OST and Anatomy-Guided techniques, respectively. The incidence rate of OST could be caused by the intra-sinus path and lack of surrounding bone due to the palatal entrance. In recent systematic reviews, the frequency of this complication varies between 1.5 and 7.5% [17, 20]. One study found fistula formation in 3 patients with no persisting fistula after a 3-year follow-up. It was suggested that modification of the palatal design of the implant could avoid potential risks for fistula formation [28]. However, in another study, 31% of cases showed a fistula, which created communication from the oral cavity into the antrum. The authors also believed that placing ZI too palatal might have caused a lack of osseointegration at the marginal level in the palatal area [30]. This affected the loading function, resulting in transversal mobility of the long coronal part of the ZI. In general, avoiding extensive countersinking preparation and fracturing the thin alveolar bone during the ZI installation is recommended to preserve the remaining bone volume as much as possible.

### Paresthesia

Seven cases of temporary paresthesia were reported in a comparative study with OST [2]. One case still presented the symptom after a 1-year follow-up, and two patients with cheekbone area hypoaesthesia were detected without mentioning if it recovered or stayed permanently [9]. The reason for post-operative paresthesia could result from intra-operative overstretching to expose the zygomatic area. A systematic review reported 15 cases of paresthesia from an affection of infraorbital and zygomaticofacial nerves [47]. Postoperative edema may also lead to temporary numbness in these areas, which may recover on its own within a short period.

### Direct surgical complications

Since surgical complications were rarely discussed in all clinical studies, both techniques may underestimate numbers. The most reported one is facial hematoma after an operation due to the broad surgical field exposure in the zygomatic arch and zygomatic area. Lip laceration has also been observed after the operation, as the limited mouth opening and long drill of ZI could damage the lips without appropriate protection. Patients with lower jaw dentition or small mouth openings should know that the drilling procedure may be more complicated than for edentulous patients. Two cases of orbital penetration and infection were described as [40, 41], resulting in conjunctiva hematoma [40]. Four studies used computer-guided surgery to place ZI, 2 with static surgical template-assisted, and the other 2 with real-time navigation [4, 14, 36, 44]. In Stievenart’s study, one static template failed to position in the correct position, leading to 3 ZIs being placed in malposition and failing in the early stage [36]. In Wu’s study, 188 ZIs were placed with a 98.4% survival rate following real-time navigation. The study showed a promising result of planned/placed accuracy [44]. Similar results were also evaluated in one systematic review, which included 12 articles with 150 ZI inserted with the help of a computer-aided navigation approach [56]. However, future investigation needs to be continued to verify the long-term feasibility.

### Prosthetic complications

Complications relating to the prosthetic restoration were reported for 56 and 104 cases in OST and Anatomy-Guided, including abutment screw loosening, abutment screw fracture, framework fracture, occlusal surface fracture, and esthetic problems. Most results were drawn from Aparicio’s study, in which the fracture of the occlusal surface of acrylic and porcelain was the most observed complication [19]. For OST and its emergence more palatal when compared to the natural dentition, bulky restorations from the abutment connection at the palatal aspect were commonly reported. Four of 13 OST studies mentioned this problem, which might lead to upholding hygiene and speech discomfort compared to conventional restorations [19, 28–30]. The Anatomy-Guided concept aims to accomplish a prosthetically driven implant trajectory that places the implant head at the natural dental position at the alveolar level or as near as possible [37].

### Limitations of the review and future research

A limitation of the present systematic review is that it fails to conduct a meta-analysis between the eligible studies. Despite efforts to homogenize study selection, all the studies had different study types, number of patients, observational periods, and lack of clear surgical procedure descriptions, which made a statistical comparison or additional subgroup analyses in reporting impossible. Future standardized studies should be contributed to assess comparable data for the clinical measurements. For the dynamic computer-assisted ZI surgery, future studies need to have larger sample sizes and long-term results for the evaluation.

## Conclusion

The conclusions drawn in this systematic review must be interpreted cautiously because of the large heterogeneity in study designs and the limited number of eligible studies/study groups per topic. Though based on the current systematic review to place ZI for rehabilitating severely atrophic edentulous maxillae with the OST and Anatomy-Guided technique, both are associated with a high implant survival rate and a low rate of surgical complications. Here, sinusitis and soft tissue infection around the implant are the most reported. However, numbers might be underestimated in both techniques since most studies have yet to report the presence or absence of complications. Both immediate and delayed protocols are described with a high implant survival rate. The utilization of immediate loading protocol is more observed in the Anatomy-Guided technique than in OST.

## Data Availability

Not applicable.
